# *Scutellaria baicalensis*, the golden herb from the garden of Chinese medicinal plants

**DOI:** 10.1007/s11434-016-1136-5

**Published:** 2016-07-08

**Authors:** Qing Zhao, Xiao-Ya Chen, Cathie Martin

**Affiliations:** 1 Plant Science Research Center, Shanghai Chenshan Botanical Garden, Shanghai Key Laboratory of Plant Functional Genomics and Resources, Shanghai, 201602 China; 2 Department of Metabolic Biology, John Innes Centre, Norwich, NR4 7UH UK; 3 Institute of Plant Physiology and Ecology, Shanghai Institutes for Biological Sciences, Chinese Academy of Sciences, Shanghai, 200032 China

**Keywords:** *Scutellaria baicalensis*, Flavonoids, Anti-cancer, Metabolic biology, Medicinal plants

## Abstract

*Scutellaria baicalensis* Georgi, or Chinese skullcap, has been widely used as a medicinal plant in China for thousands of years, where the preparation from its roots is called Huang-Qin. It has been applied in the treatment of diarrhea, dysentery, hypertension, hemorrhaging, insomnia, inflammation and respiratory infections. Flavones such as baicalin, wogonoside and their aglycones baicalein wogonin are the major bioactive compounds extracted from the root of *S. baicalensis*. These flavones have been reported to have various pharmacological functions, including anti-cancer, hepatoprotection, antibacterial and antiviral, antioxidant, anticonvulsant and neuroprotective effects. In this review, we focus on clinical applications and the pharmacological properties of the medicinal plant and the flavones extracted from it. We also describe biotechnological and metabolic methods that have been used to elucidate the biosynthetic pathways of the bioactive compounds in *Scutellaria*.

## 摘要

黄芩是一种常用的药用植物, 中国人对它的使用了已有数千年历史。黄芩根的制备物, 为常用的中药材, 在中国传统医学中用来治疗腹泻、痢疾、高血压、出血、 失眠、炎症和呼吸道感染。黄芩根中主要的活性物质为黄酮物质黄芩苷, 汉黄芩苷及 苷元黄芩素, 汉黄芩素。药理学研究显示这些黄酮物质具有多种药物学活性, 包括抗 癌, 保肝、抗菌、抗病毒、抗氧化、抗惊厥和神经系统保护作用。在本综述里面, 我 们集中介绍了黄芩的临床应用及药物学活性。我们也介绍了用于研究黄芩中活性物质 代谢途径的生物技术及代谢生物学方法。

## Introduction

**Fig. 1:**
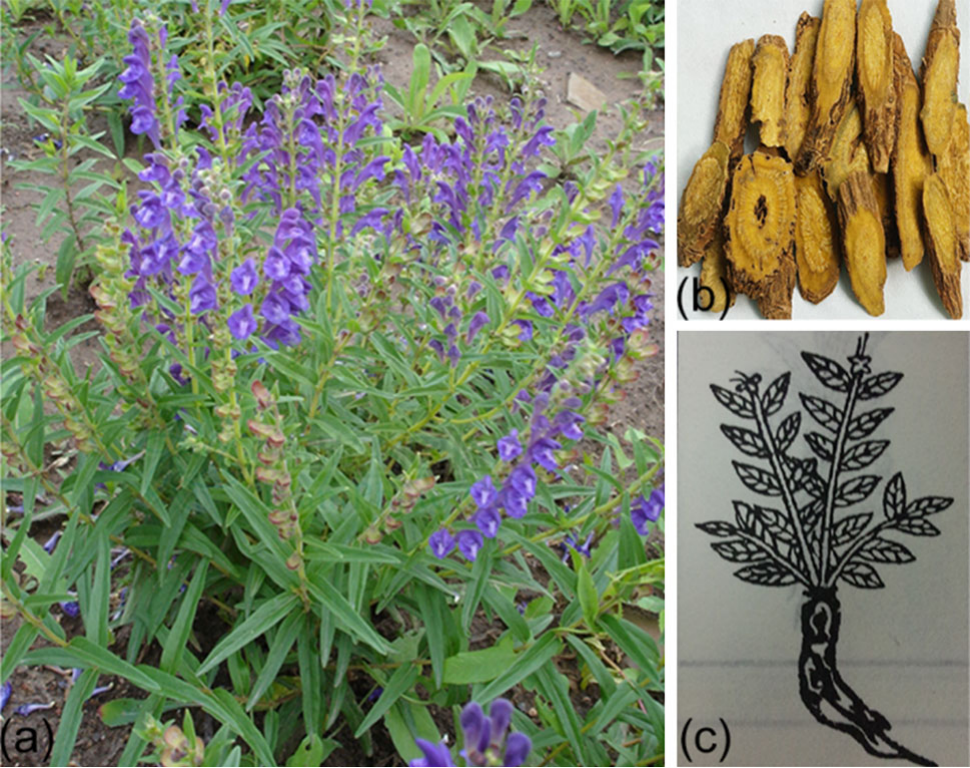
(Color online) The medicinal plant *Scutellaria baicalensis*, known as Huang-Qin. **a** *Scutellaria baicalensis* Georgi plant. **b** The dried root of *S. baicalensis* used in traditional Chinese medicine. **c** A hand-drawn figure of *S. baicalensis* in *Bencao Gangmu* (*Compendium of Materia Medica*) by Li Shizhen

*Scutellaria baicalensis* Georgi is a species of flowering plant in the Lamiaceae family (Fig. 1a). It is indigenous to several East Asian countries and the Russian Federation and has been cultivated in many European countries [1, 2]. Chinese people have used the dried root of this medicinal plant for more than 2000 years as a traditional medicine known as Huang-Qin (Fig. 1b) and it is now listed officially in the Chinese Pharmacopoeia. The dried root of Huang-Qin is often prepared by decoction (boiling) or as tinctures [3]. Huang (黄) means yellow. Qin (芩) is equivalent to Jin (菳), and means golden herb, as explained in *Shuowen Jiezi*, an early 2nd-century Chinese dictionary from the Han Dynasty [4, 5]. Huang-Qin was first recorded in *Shennong Bencaojing* (*The Classic of Herbal Medicine*), written between about 200 and 250 AD, for treatment of bitter, cold, lung and liver problems [6]. The most authoritative book on traditional Chinese medicine, *Bencao Gangmu* (*Compendium of Materia Medica*) which was first published in 1593, reported that *Scutellaria baicalensis* (Fig. 1c) had been used in the treatment of diarrhea, dysentery, hypertension, hemorrhaging, insomnia, inflammation and respiratory infections. Its author, Li Shizhen, reported successful self-administration to treat a severe lung infection when he was 20 years old [4].

## Clinical applications

*Scutellaria baicalensis* has been used as a medicine in several East Asian countries for more than 2000 years. Clinical data for this herb are accumulating and Huang-Qin alone has been reported to be useful for treating colds and bacterial pneumonia [7, 8].

In many Eastern countries, Huang-Qin is prescribed as a part of a multi-herb formulation. Huang-Qin is an important ingredient of Xiaochai Hutang (Chinese) or Sho-saiko-to (SST, Japanese) preparations, first described in *Shanghan Lun* (On Cold Damage), written by Zhang Zhongjing around 200 AD [9]. This formulation was described as having ‘worked effectively in some instances where conventional Western therapies failed or proved to be insufficient to provide a palliative cure’ by Xue and Roy in 2003 [10] and was subsequently taken up by the alternative medicine community in the USA [11]. A study of the effects of SST on hepatitis was reported by a Japanese group in 1994 [12]. Ninety-eight hepatitis patients were treated with SST and followed up for 5 years. Liver function was improved in 78 % of the hepatitis B patients and in 67 % patients with non-A non-B type hepatitis, with significantly reduced serum levels of aminotransferase AST, ALT, and rGTP [12]. SST is also effective in hepatitis C patients. Eighty hepatitis C patients who were interferon-resistant were treated with SST combined with a common unspecified medicine or the common medicine alone. These patients were studied for 7 years during which time, 5 patients on the SST treatment achieved fully normalized enzyme functions. Liver enzyme normalization was observed in only one control patient. Conversely, 5 control patients (common medicine alone) progressed to liver cancer compared to just one on the SST combination therapy [13].

Lung Fufang, another traditional prescription using Huang-Qin, can prolong the survival rate of patients with primary bronchial pulmonary squamous cell carcinoma [14], and it has a similar effect on NSCLC (non-small-cell lung cancer) patients. Elderly people suffering from NSCLC and treated with Lung Fufang Prescription showed improved indices for the clinical syndrome and improved quality of life compared to the control group who were treated with normal chemotherapy plus a TCM (Traditional Chinese Medicine) placebo [15]. Huang-Qin is also a major ingredient of Fuzheng anti-cancer prescription, which has been used in combination with chemotherapy and shown to have improved outcomes on NSCLC in middle and late stage patients, compared to conventional chemotherapy alone [16].

## Pharmacology of Huang-Qin

### Antitumor effects

Many studies have shown that *S. baicalensis* extract is cytotoxic to a broad range of cancer cells from humans, including brain tumor cells [17], prostate cancer cells [18] and HNSCC (head and neck squamous cell carcinoma) cell lines [19]. Aqueous extracts of *S. baicalensis* roots induced apoptosis and therefore suppressed growth of lymphoma and myeloma cell lines, by changing the expression levels of *Bcl* genes, increasing cyclin-dependent kinase inhibitor p27 (KIP1) activity and decreasing expression of the c-myc oncogene [20]. Similarly, *S. baicalensis* extracts were selectively toxic to several human lung cancer cell lines, but not to normal human lung fibroblasts. Increases in p53 and Bax protein activities may be responsible for these effects [21].

The flavones baicalin, wogonoside and their aglycones baicalein and wogonin are the major bioactives in Scutellaria roots and the major bioactive constituents responsible for anti-cancer effects of Huang-Qin [22–24]. Baicalin inhibits growth of lymphoma and myeloma cells [20]. Wogonoside has anticancer effects on acute myeloid leukemia (AML) cell lines and on primary patient-derived AML cells. It increases significantly the transcription of phospholipid scramblase 1 (PLSCR1), a regulator of the cell cycle and differentiation-related genes [25]. Baicalin, baicalein and wogonin have similar effects as *S. baicalensis* extracts against lung cancer cells [21]. The anti-cancer activities of the *Scutellaria*-derived flavones have been mainly ascribed to their ROS scavenging ability, attenuation of NF-κB activity, cell cycle gene expression, COX-2 gene expression and prevention of viral infections [22, 26, 27].

In a high-throughput screen of over 4000 compounds to detect genotoxic compounds using a quantitative cell-based assay, Fox et al. [28] identified 22 antioxidants, including baicalein. Treatment of dividing cells with baicalein induced DNA damage and resulted in cell death. Despite this genotoxic effect, baicalein did not induce mutations, a major problem of conventional anticancer drugs, suggesting that baicalein and related flavones are strong candidates for improved chemotherapeutic agents [28].

### Hepatoprotection

*Scutellaria baicalensis* is the main component in the herbal remedy SST used for liver problems such as hepatitis, hepatic fibrosis and carcinoma [11, 29, 30]. Yang-Gan-Wan (YGW) is another prescription containing baicalin, which has long been known for its protective effects on the liver [31, 32]. This herbal prescription prevents and reverses activation of hepatic stellate cells, (HSC; the major pathogenic cell type in fibrogenesis) by epigenetic derepression of PPARγ (Peroxisomal proliferator-activated receptor γ), so preventing liver fibrosis. Baicalin is a major active phytocompound in Yang-Gan-Wan (YGW) and suppresses the expression and signaling by canonical Wnts, which are involved in epigenetic repression of PPARγ [33].

Several studies have suggested that *S. baicalensis* can effectively inhibit fibrosis and lipid peroxidation in rat liver [34–36]. Consumption of the roots and shoots of *S. baicalensis* inhibits mutagenisis caused by the aflatoxin-B1 mycotoxin in rat liver cells [35]. The anti-fibrosis activity of *S. baicalensis* root extracts may be due to enhanced phosphorylation of the cAMP response element binding protein as proposed by Tan et al. [37], although extracts of *Scutellaria baicalensis* roots also arrest the cell cycle, activate the caspase system and activate ERK-p53 pathways resulting in apoptosis of HSC-T6 cells to prevent hepatic fibrosis [38].

### Antibacterial and antiviral activities

Amongst 46 herb and spice extracts, *S. baicalensis* extracts have shown substantial antibacterial effects against *Bacillus cereus*, *Escherichia coli, Listeria monocytogenes*, *Salmonella anatum* and *Staphylococcus aureus* [39]. Aqueous extracts of *S. baicalensis* roots have antimycotic properties against *Aspergillus fumigatus*, *Candida albicans*, *Geotrichumcandidum* and *Rhodotorula rubra* [40]. Baicalin, isolated from *S. baicalensis*, has been applied as a natural antibacterial agent against foodborne pathogens such as *Salmonella* and *Staphylococcus* spp. in homemade mayonnaise [41]. Extracts of *S. baicalensis* can also enhance the antimicrobial activity of several antibiotics such as ciprofloxacin, ceftriaxone, gentamicin and penicillin G, against *Staphylococcus aureus* [42].

Xiaochai Hutang or Sho-saiko-to (SST) is effective against hepatitis, and a reduction of viral load has been observed in some patients treated with SST [11], indicating an antiviral function of Scutellaria extracts [43]. Scutellaria root extracts can inhibit the replication of HCV-RNA significantly [44].

Baicalin has very good anti-HIV-1 activity as a non-nucleoside reverse transcriptase inhibitor [45]. Moreover, baicalin can prevent the entry of HIV-1 into animal cells by perturbing the interaction between HIV-1 Env and HIV-1 co-receptors on the cell surface [46]. Baicalin has been adopted as one of the popular lead natural products for preventing HIV infection [47]. Differences in the inhibitory activities of baicalein and baicalin against HIV-1 reverse transcriptase have been evaluated by Zhao et al. [48]. They found that baicalein has four times stronger inhibitory activity on HIV-1 reverse transcriptase than baicalin. However, baicalin can be deglycosylated to form baicalein in the human body [48].

Aqueous extracts of *S. baicalensis* elicit significant inhibition (91.1 %) of HIV-1 protease activity at concentrations of 200 µg/ml [49]. Early in 1989, Ono et al. [50] reported baicalein could effectively inhibit reverse transcriptase activity of human immunodeficiency virus (HIV); 2 μg/mL baicalein inhibiting 90 % of the activity of HIV reverse transcriptases [50]. Baicalein is also an inhibitor of HIV-1 integrase, an essential enzyme in the life cycle of the virus, by binding to the hydrophobic region of the HIV-1 integrase catalytic core domain to induce a conformational change [51]. These effects of baicalein and baicalin on HIV have attracted considerable attention [52].

### Other effects

In addition to the effects described above, preparations of *S. baicalensis* can also work as antioxidants, ROS scavengers [53, 54] and anticonvulsants [55]. Recently, the neuroprotective effects of *S. baicalensis* and its component flavones, have been studied using both *in vitro* and *in vivo* models of neurodegenerative diseases. Results suggest that this medicinal plant may have promising applications in neuroprotection [56, 57].

## Biotechnology to enhance *S. baicalensis* synthesis

**Fig. 2:**
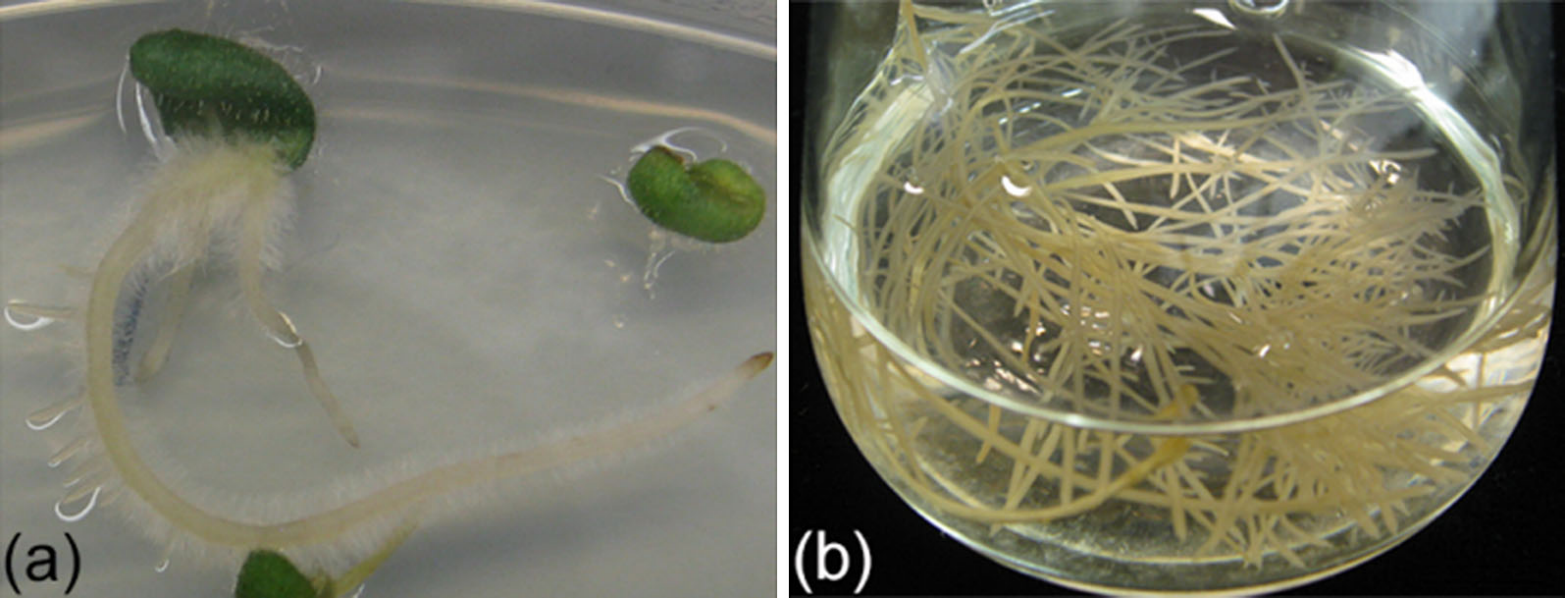
(Color online) Production of hairy root cultures of *Scutellaria baicalensis*. **a** Hairy roots induced by infection of a cotyledonary explant of *S. baicalensis* by *Agrobacterium rhizogenes*. **b** Liquid culture of Scutellaria hairy roots

Given their established bioactivity, the possibility to enhance production of the flavones in this plant or alternatively produce them in common vegetables or fruits is attractive [58, 59]. Understanding the regulation of production of bioactive flavones (baicalein, baibalin, wogonin and wogonoside) and their biosynthesis in *S. baicalensis*, and developing strategies to enhance their production are important objectives. However, like other members of the mint family, stable genetic transformation and regeneration of this plant are very difficult. *Agrobacterium rhizogenes*-mediated production of hairy roots of *S. baicalensis* has proved to be effective in this recalcitrant species [60, 61] (Fig. 2). Hairy roots can be induced from either leaf or cotyledon explants [62, 63] in an *A. rhizogenes* strain-dependant manner. Among the four strains (A4GUS, R1000 LBA 9402 and ATCC11325) tested by Tiwari et al.(2008), the A4 stain produced the most hairy roots, with an efficiency of 42.6 % [60]. Supplementation of acetosyringone during co-cultivation of plant tissue and *A. rhizogenes* enhanced the transformation efficiency further [64]. Hairy root cultures of *S. baicalensis* have a similar metabolite pattern to natural roots and the major flavones can be enhanced by treatment of cultures with methyl jasmonate [65–67]. Over-expression of PAL or CHI in hairy roots of Scutellaria leads to enhanced levels of root-specific flavones [63, 68] (Table 1).

**Table 1:** Composition of multi-herb formulations containing *S. baicalensis*

Name	Compositions	References
Xiaochai Hutang	*Scutellaria baicalensis*, *Bupleurum falcatum*, *Pinellia ternate*, *Panax ginseng*, *Glycyrrhiza uralensis*, *Zingiber officinale*, *Ziziphus jujuba*	[9, 11]
Lung fufang	*Panax ginseng*, *Astragalus* *membranaceus*, *Lycium* *barbarum*, Glossy privet fruit (*Ligustrum lucidum*), Sichuan fritillary bulb (*Fritillaria cirrhosa*), Radix Ophiopogonis (*Ophiopogon japonicus*), *Platycodon* *grandiflorum*, *Scutellaria baicalensis*, Lily bulb (*Lilium brownii*), *Curcuma* *zedoary*, pseudo-ginseng (*Panax notoginseng*), *Oldenlandia* *diffusa*	[14, 15]
Fuzheng anti-cancer prescription	*Astragalus* *membranaceus*, American ginseng (*Panax quinquefolius*), *Citrus reticulate*, *Pinellia ternate*, *Scutellaria baicalensis*, *Poria* *cocos*, *Atractylodes Lancea,* *Schisandra* *chinensis*, *Oldenlandia* *diffusa*, *Adenophora* *stricta*, *Salvia miltiorrhiza*	[16]

Next-generation sequencing technologies have been employed to screen for candidate genes that may be responsible for biosynthesis of the flavones, and several structural genes including 6-hydroxylase, 8-O-methyltransferase, 7-O-glucuronosyltransferases have been suggested to be involved in their biosynthesis [69]. Yuan et al. [70, 71] also screened RNA-sequencing databases and found that several MYB genes may be responsible for regulation of production of its flavonoids.

## Flavonoid metabolism

**Fig. 3:**
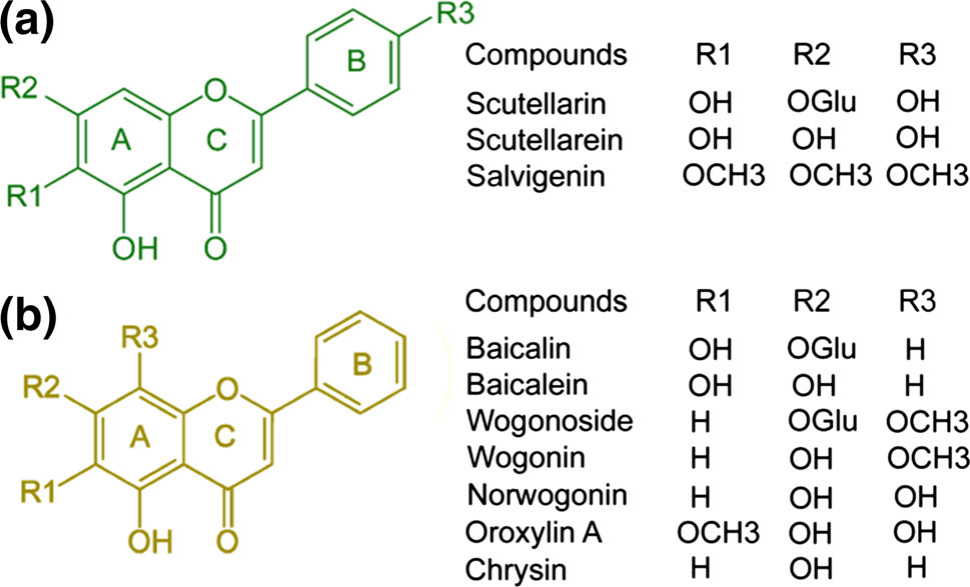
(Color online) Major flavones in *Scutellaria baicalensis*. **a** Flavones produced from naringenin. **b** Root-specific 4′-deoxyflavones, originating from pinocembrin

*Scutellaria baicalensis* Georgi produces various natural products including amino acids, essential oils, flavonoids, phenylethanoids, and sterols. More than 30 types of flavones can be found in its roots (Fig. 3), including baicalin, baicalein, chrysin, oroxylin A, oroxylin A 7-O-glucuronide, wogonin and wogonoside [72, 73]. Baicalin, baicalein, wogonin, and wogonoside are the major bioactive compounds extracted from *S. baicalensis* Georgi [74–76].

**Fig. 4:**
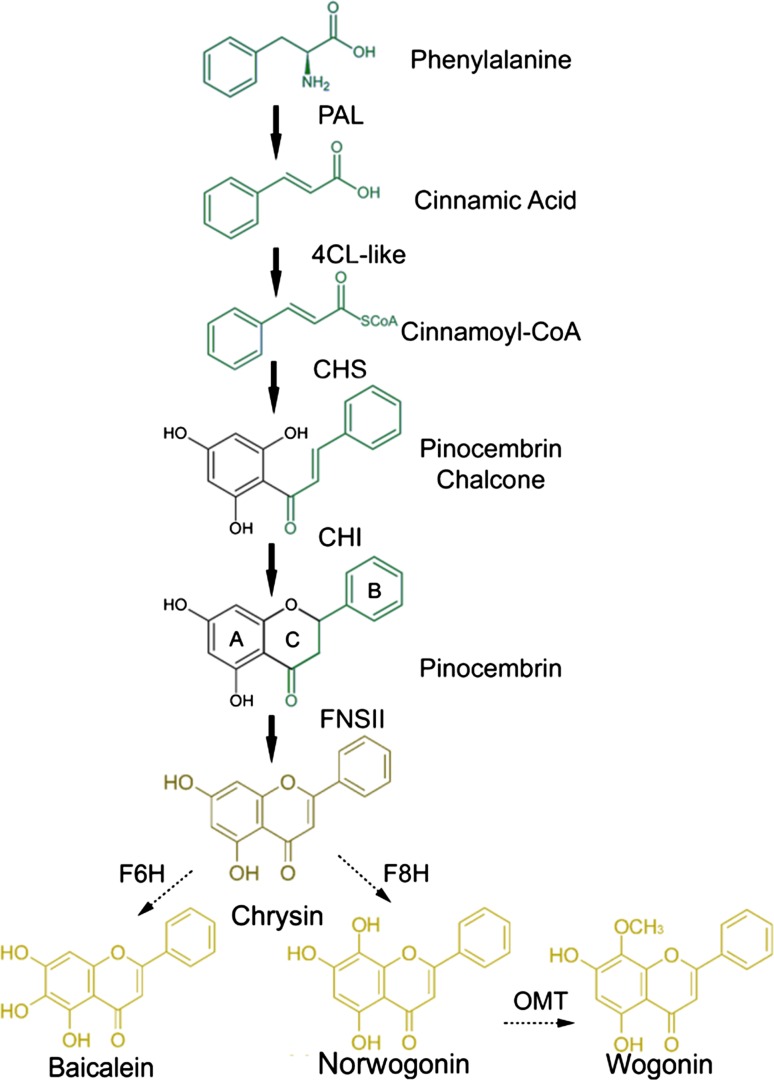
(Color online) The proposed biosynthetic pathway for production of root-specific flavones of *Scutellaria*

Flavones are present in aerial tissues of many flowering plants, with roles in co-pigmentation of flowers (they make anthoyanin pigments appear bluer) and in protection against UV irradiation [77, 78]. Flavones are synthesized by the flavonoid pathway, which is part of phenylpropanoid metabolism [79, 80]. Naringenin is a central intermediate in normal flavone biosynthesis [81] exemplified by the production of the flavones, scutellarin and scutellarein, derived from naringenin in the aerial parts (leaves and flowers) of *Scutellaria baicalensis*. Scutellarein and scutellarin are synthesised from phenylalanine by general phenyl propanoid metabolism; phenylalanine ammonia lyase (PAL), cinnamoyl 4 hydroxylase (C4H) and p-coumaroyl CoA ligase (4CL) followed by chalcone synthase (CHS) and chalcone isomerase (CHI) to form naringenin [82]. A flavone synthase (FNSII-1) then oxidises naringenin to form apigenin, which may be further hydroxylated, methylated and glycosylated to form scutellarein and scutellarin (Fig. 3a). Scutellaria roots however accumulate large amounts of specialized root-specific flavones (RSFs), lacking a 4′-OH group on their B-rings (Fig. 3b) [83]. These RSFs, which include baicalein and wogonin, and their glycosides, are not synthesized from naringenin, but by an alternative pathway where cinnamic-acid is recruited by a specially-evolved cimmamoyl-CoA ligase (SbCLL-7) to form cinnamoyl CoA which is then condensed with malonyl CoA by a specialised isoform of chalcone synthase (SbCHS-2) to form a chalcone, which is then isomerized by the same chalcone isomerase (CHI) that acts in scutellarin biosynthesis, to form pinocembrin, a flavanone without a 4′-OH group. Pinocembrin is converted by a specialised isoform of flavone synthase (FNSII-2), to form chrysin, which serves as the founding 4′ deoxyflavone which may be decorated further by 6/8-flavone hydroxylases, 8-O-methyl-transferases and glycosyltransferases to produce the different RSFs produced in the roots of *S. baicalensis* [64, 84] (Fig. 4).

The evolution of this specialised pathway for 4′ deoxyflavone biosynthesis occurred relatively recently, following the divergence of the Laminaceae [64] and may have been facilitated by the recruitment of a CoA ligase activity from a gene encoding an enzyme of fatty acid metabolism, that is specific for cinnamate. Effective competition for cinnamate in the face of high level expression of C4H may have paved the way for effective production of 4′- deoxyflavones in roots of *S. baicalensis*. Production of 4′- deoxyRSFs in roots is induced by methyl jasmonate treatment, suggesting that RSFs are made as part of a defence mechanism or for plant–microbe signalling [85, 86]. Understanding the regulation of this newly-evolved pathway may facilitate engineering of biosynthesis of these important bioactive metabolites. Their roles in defence in Scutellaria may also underpin some of their uses in traditional medicine, for example as anti-microbials.

The bioactive compounds baicalein, wogonin and their glysosides can be found in many species from the genus Scutellaria other than *S. baicalensis* [87]. As in traditional Chinese medicine, the roots of *S. amoena* and *S.likiangensis* have been used commonly as alternatives to *S. baicalensis.* To date, 4′-deoxyflavones have been found only in *Oroxylum indicum* vent [88] and *Plantago major* L. outside the genus *Scutellaria* but in the order Lamiales [89]. 4′-Deoxyflavones have also been reported in *Anodendron affine* and *Cephalocereus senilis* outside the order Lamiales [90, 91]. The evolution of metabolic pathways determining the taxa-specific distribution of these 4′-deoxyflavones is fascinating, and we suspect that convergent evolution has most likely influenced the development of metabolic pathways responsible for producing these specialised bioactive flavones in widely diverged plant species [92, 93].
